# Association Between Diet, Physical Activity, Smoking, and Ultra-Processed Food and Cardiovascular Health, Depression, and Sleep Quality

**DOI:** 10.7759/cureus.66561

**Published:** 2024-08-10

**Authors:** Fernanda Maltos-Gómez, Azucena Brito-López, Julián B Uriarte-Ortiz, Diana P Guízar Sánchez, Armando Muñoz-Comonfort, Raúl Sampieri-Cabrera

**Affiliations:** 1 Physiology, Facultad de Medicina, Universidad Nacional Autónoma de México, México City, MEX; 2 Prospective, Centro de Ciencias de la Complejidad, Universidad Nacional Autónoma de México, México City, MEX

**Keywords:** cardiovascular health, physical activity, diet, sleep quality, depression, young adults

## Abstract

Background: This study evaluated cardiovascular health, dietary habits, physical activity, depression, and sleep quality in young university adults.

Materials and methods: A cross-sectional design was used to assess anthropometric, biochemical, and cardiovascular health behaviors. The study included 158 university students aged 18 to 30 years (65% women, 35% men, average age: 20.3 ± 2.4 years), selected through non-probabilistic sampling. Measurements included BMI, waist circumference, blood pressure, glucose, triglycerides, HDL and LDL cholesterol, and visceral fat using bioelectrical impedance. Health behaviors were evaluated via questionnaires on physical activity, fruit and vegetable consumption, smoking, ultra-processed food consumption, and sleep quality using the Pittsburgh Sleep Quality Index. The cardiovascular health index was assessed with the "Life's Essential 8" questionnaire and depression was assessed with Beck Depression Inventory. Statistical analyses included ANOVA, Fisher's F test, Student's t-test, and simple linear regression, conducted using SPSS Statistics version 25.0 (IBM Corp. Released 2017. IBM SPSS Statistics for Windows, Version 25.0. Armonk, NY: IBM Corp), with significance set at p<0.05.

Results: Women showed better adherence to healthy behaviors. Higher fruit and vegetable consumption and physical activity were associated with lower visceral fat. Higher visceral fat is correlated with increased blood pressure and decreased HDL cholesterol. Smoking and frequent ultra-processed food consumption were linked to higher depression scores, which were associated with poorer sleep quality.

Conclusion: Healthy lifestyle habits are crucial for physical and mental health, providing a basis for public health interventions.

## Introduction

Chronic degenerative diseases (CDDs) are one of the greatest challenges for modern public health [1]. CDDs are responsible for more than 70% of all deaths worldwide [2]. Low- and middle-income regions are the most affected, and this is exacerbated by the scarcity of healthcare resources [3]. Additionally, socioeconomic, cultural, and environmental factors contribute to the high prevalence of these diseases [4]. Furthermore, lifestyle changes driven by urbanization and globalization have led to an increase in the incidence of obesity, diabetes, and cardiovascular diseases [5]. To address this public health challenge, health education, access to quality healthcare services, and public policies focused on prevention appear to be key in reducing the mortality and morbidity associated with these diseases [6-8]. CDDs develop slowly but with a constant progression. This process can take years, during which initial symptoms may be mild or even nonexistent, making early detection a challenge [9]. Diabetes mellitus, for example, is when blood glucose levels remain elevated due to insufficient insulin production or tissue resistance to this hormone. Without proper management, it can lead to serious complications such as cardiovascular diseases, nerve damage, and kidney problems [10]. Hypertension is characterized by persistently elevated blood pressure. This condition increases the risk of developing cardiovascular diseases such as heart attacks and strokes [11]. Similar to diabetes, cardiovascular diseases, which encompass a variety of conditions affecting the heart and blood vessels, are the leading cause of death worldwide [12]. For example, the obstruction of coronary arteries can lead to myocardial infarctions, while the accumulation of plaque in the arteries can cause strokes [13]. These diseases are interrelated with other risk factors such as smoking, unhealthy diet, physical inactivity, and excessive alcohol consumption [14]. Therefore, CDDs require continuous management, which affects the quality of life of patients and has implications at physical, economic, social, and mental levels [15]. Additionally, the burden they place on healthcare systems is enormous, as they require significant resources for long-term management and treatment [16]. The impact of CDDs goes beyond direct medical costs; it includes loss of productivity, disabilities, and, in many cases, premature death [17].

The prevention of CDDs represents a fundamental pillar of public health. Primary prevention focuses on preventing the onset of disease by promoting healthy lifestyles. This includes interventions such as promoting physical activity, nutritional education, awareness campaigns on the risks of smoking and alcohol, and fostering mental health [18]. A good primary prevention strategy includes the concept of cardiovascular health, defined by the American Heart Association (AHA) as the ability of the heart and blood vessels to work in harmony with the rest of the body, thus ensuring optimal function and longevity. To calculate a cardiovascular health index, the AHA establishes the "Life's Essential 8" as a guide to evaluate and improve cardiovascular health [19]. These include a healthy diet (balanced nutrition rich in fruits, vegetables, whole grains, lean proteins, and healthy fats), physical activity (at least 150 minutes of moderate or 75 minutes of vigorous activity per week), healthy weight (maintaining a BMI within the normal range), no smoking (avoiding tobacco products and secondhand smoke exposure), blood pressure (keeping blood pressure below 120/80 mmHg), cholesterol (maintaining total cholesterol levels below 200 mg/dL), blood glucose (keeping fasting blood glucose below 100 mg/dL), and sleep duration and quality (sleeping between seven and nine hours per night with good sleep quality). These eight components are essential for preventing cardiovascular diseases and promoting a long and healthy life. Adopting these habits contributes to better overall health and a lower incidence of adverse cardiac events at any stage of life [20-22].

In the young adult population, aged 18 to 30 years, preventing the development of cardiovascular diseases is crucial due to several factors influencing both individual and public health. This stage of life is key for establishing health habits that will persist over time [23,24]. During these years, individuals tend to form behavioral patterns related to diet, physical activity, and stress management [20,25]. Moreover, the impact of preventing cardiovascular diseases in the young population extends beyond individual health, affecting the well-being of the community at large. A healthy young population is more productive and can contribute more effectively to economic and social development. Additionally, promoting cardiovascular health in young adults can have a multiplier effect, influencing their peers and future generations through education and example.

In this regard, the present cross-sectional study was conducted with a sample of young university adults by evaluating various anthropometric and biochemical parameters, as well as cardiovascular health behaviors. The primary aim was to understand their cardiovascular health status and assess the association between factors and components in cardiovascular health status.

## Materials and methods

Study design

We conducted a cross-sectional study to evaluate anthropometric, biochemical, and cardiovascular health behavioral parameters in a sample of adult participants. Data were collected using self-administered questionnaires and direct measurements in a controlled environment. This study was approved by the Research Division of the School of Medicine at the Universidad Nacional Autónoma de México (approval number: FM/DI/022/2021).

Participants

The study sample consisted of male and female university students aged between 18 and 30 years. The participants were selected through non-probabilistic sampling. Only those who voluntarily agreed to participate in the study and signed the informed consent form were included.

Anthropometric and biochemical assessments

The anthropometric and biochemical assessments were conducted as follows: BMI was calculated using the formula weight in kilograms divided by the square of height in meters. Waist circumference was measured at the midpoint between the lower margin of the last palpable rib and the top of the iliac crest using a flexible tape measure. Blood pressure readings, including systolic and diastolic values, were obtained using a calibrated sphygmomanometer, following standard protocols. For biochemical measurements, capillary blood samples were collected using a lancet and analyzed for glucose, triglycerides, and HDL and LDL cholesterol levels using standardized enzymatic methods. Visceral fat was quantified with a bioelectrical impedance analyzer (InBody 270), which estimates fat distribution by measuring body resistance to electrical currents. This process was conducted under controlled conditions to ensure the accuracy and reliability of the data.

Health behaviors

Health behaviors were evaluated using a questionnaire that included questions on (1) physical activity (frequency and weekly duration), (2) fruit and vegetable consumption (quantity and weekly frequency), (3) smoking habits (presence and quantity), (4) consumption of ultra-processed foods (weekly frequency), and (5) sleep quality (assessed using the Pittsburgh Sleep Quality Index (PSQI), Appendices [26]).

Health index

Ideal cardiovascular health index (assessed with the "Life's Essential 8" questionnaire from the AHA [19], Appendices) and Beck Depression Inventory (BDI was used to evaluate the emotional well-being of participants, Appendices [27]).

Statistical analysis

ANOVA, Fisher's F test, and Student's t-test were conducted as appropriate: (1) assessment of differences in visceral fat levels based on fruit and vegetable consumption and physical activity levels, (2) comparison of BDI-2 scores among different groups of smokers and levels of ultra-processed food consumption, and (3) evaluation of differences in PSQI scores according to depression categories. Additionally, simple linear regression analyses were performed to evaluate the relationship between visceral fat levels and cardiovascular health parameters. Descriptive analysis was used to examine the distribution of participants' nutritional status. Data analysis was conducted using SPSS Statistics version 25.0 (IBM Corp. Released 2017. IBM SPSS Statistics for Windows, Version 26.0. Armonk, NY: IBM Corp.) Statistical significance was set at p<0.05.

## Results

Sociodemographic data

The total number of participants was 158, with 65% women (n=103) and 35% men (n=55). The average age of the participants was 20.3 ± 2.4 years, with no statistically significant difference in age between women (20.2 ± 2.5 years) and men (20.4 ± 2.3 years) (p=0.76). All the participants were university students. The average monthly income was 4.5 times the minimum wage, with no significant difference between women (4.2 ± 0.5) and men (4.6 ± 0.2) (p=0.54).

Components of the cardiovascular health index by sex

**Figure 1:**
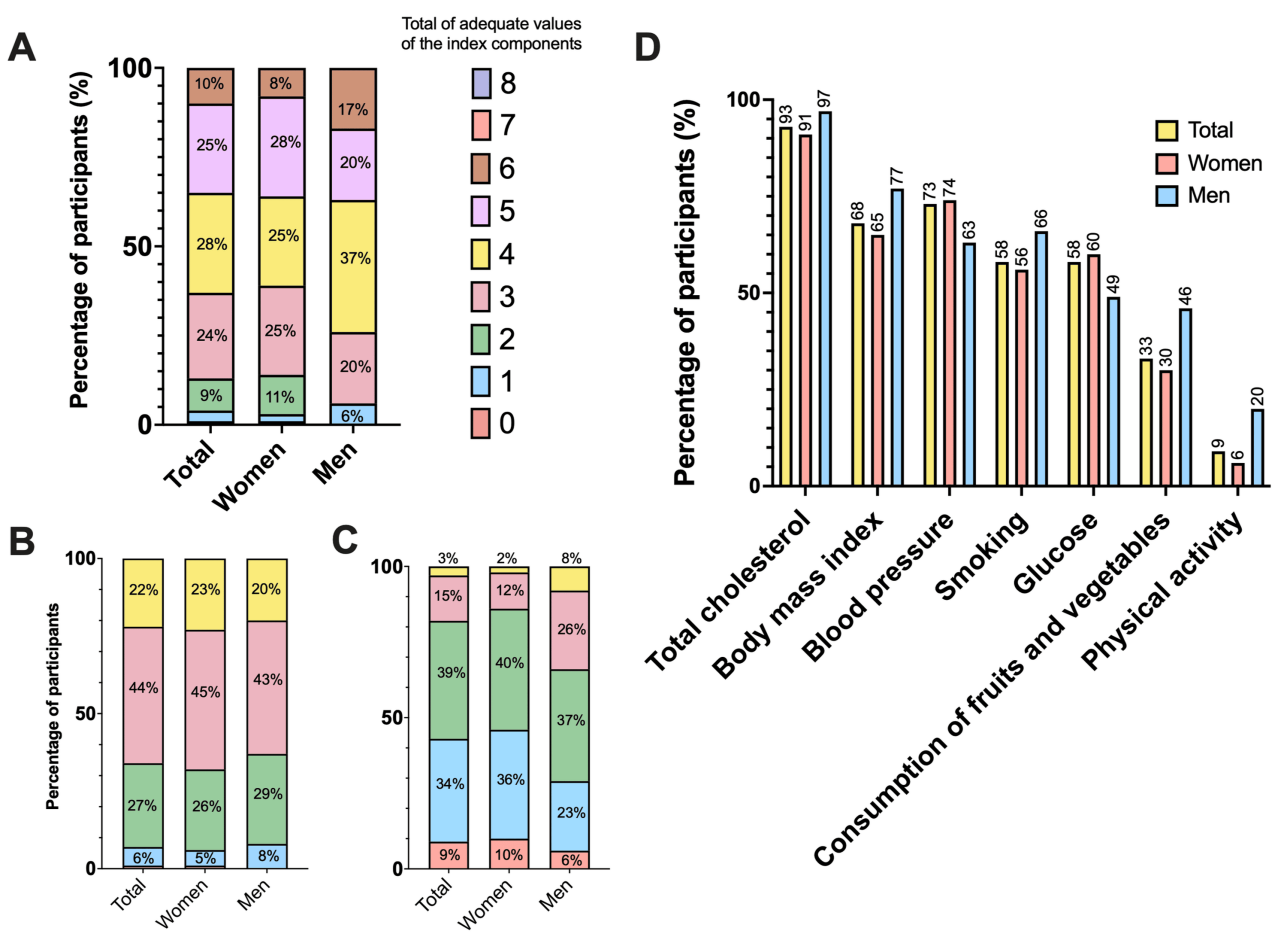
Distribution of compliance with health index components by sex (A) Percentage of total participants, women, and men according to the total number of adequately met health index components. Differences in compliance distribution are observed, with a higher percentage of men meeting three adequate components and a higher percentage of women meeting seven adequate components (p>0.05). (B) Percentage of total participants, women, and men meeting each specific health index component: total cholesterol, BMI, blood pressure, smoking, glucose, fruit and vegetable consumption, and physical activity. The data show that compliance varies between men and women for each component, with women showing higher adherence to several components, such as fruit and vegetable consumption. (C) Percentage of total participants, women, and men based on their general health status according to the number of adequately met health index components. (D) Percentage of participants meeting each specific health index component.

Women were observed to meet a greater number of components of the cardiovascular health index compared to men. Women showed better adherence to aspects such as fruit and vegetable consumption and physical activity, contributing to a more favorable health profile (Figure 1).

Impact of fruit and vegetable consumption and physical activity on visceral fat levels

**Figure 2:**
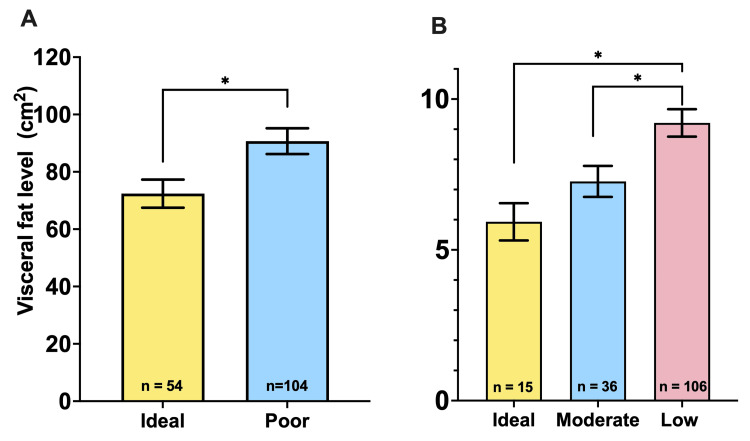
Relationship between healthy habits and visceral fat (A) Comparison of visceral fat levels between groups with ideal (n=54) and poor (n=104) fruit and vegetable consumption. Results indicate that those with adequate fruit and vegetable consumption have significantly lower levels of visceral fat. The significant difference is indicated with an asterisk (*) (p=0.012, t-value=2.55). (B) Comparison of visceral fat levels among different levels of physical activity: ideal (n=15), moderate (n=36), and low (n=106) (F=5.83, p=0.004). Results show that participants with ideal and moderate levels of physical activity have significantly lower levels of visceral fat compared to those with low levels of physical activity. Significant differences are indicated with asterisks (*) (very active vs. low activity p=0.015; moderate vs. low activity p=0.045).

Participants with higher fruit and vegetable consumption, as well as higher levels of physical activity, have higher levels of visceral fat. Participants with adequate fruit and vegetable consumption had significantly lower levels of visceral fat compared to those with inadequate consumption. Similarly, those with ideal and moderate levels of physical activity had lower levels of visceral fat than those with low levels of physical activity (Figure 2).

Correlation between visceral fat levels and cardiovascular health factors

**Figure 3:**
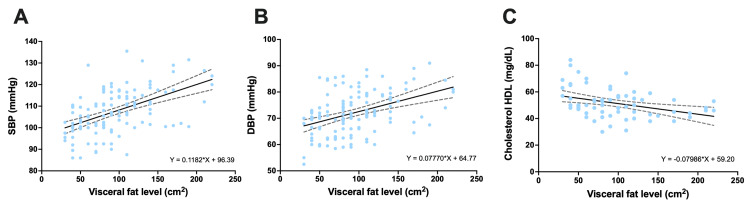
Relationship between visceral fat and cardiovascular parameters (B) Relationship between visceral fat level and SBP. A positive correlation is observed, indicating that higher levels of visceral fat are associated with higher systolic blood pressure (r=0.51, p<0.001). The linear regression equation and the 95% confidence interval are shown in the graph. (B) Correlation between visceral fat level and DBP. Similar to systolic pressure, there is a positive correlation, suggesting that higher levels of visceral fat are also associated with higher diastolic blood pressure (r=0.42, p<0.001). (C) Correlation between visceral fat level and HDL cholesterol levels. Unlike the blood pressure parameters, a negative correlation is observed, indicating that higher levels of visceral fat are associated with lower HDL cholesterol levels (r=-0.36, p=0.003). The linear regression equation and the 95% confidence interval are included in the graph. SBP: systolic blood pressure, DBP: diastolic blood pressure, HDL: high-density lipoprotein

Higher levels of visceral fat are associated with increased blood pressure and decreased HDL cholesterol levels. A significant correlation was found between visceral fat levels and various cardiovascular health parameters. Higher levels of visceral fat were associated with increased systolic and diastolic blood pressure, indicating a greater risk of hypertension. Additionally, a negative correlation was observed between visceral fat and HDL cholesterol levels, suggesting that higher levels of visceral fat are associated with lower HDL cholesterol levels, which can increase the risk of cardiovascular diseases (Figure 3).

Nutritional status of participants

**Figure 4:**
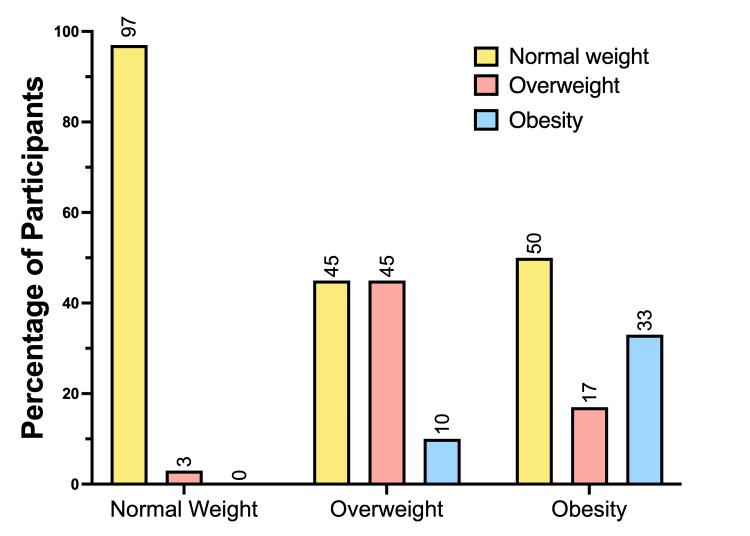
Distribution of participants' nutritional status The percentage distribution of participants is classified according to their nutritional status: normal weight, overweight, and obesity. The majority of participants fall into the normal weight category (n=68, 66.0%), with a smaller proportion in the overweight category (n=29, 28.1%) and obesity category (n=6, 5.8%). Overall, 33.9% of the sample is either overweight or obese.

The majority of participants have a normal weight, but there is a significant proportion of individuals who are overweight or obese. The majority of participants were in the normal weight category (66.0%); however, a significant proportion were overweight (28.1%) and obese (5.8%), highlighting the need for interventions to address these public health issues (Figure 4).

Effect of smoking and ultra-processed food consumption on BDI-2 scores

**Figure 5:**
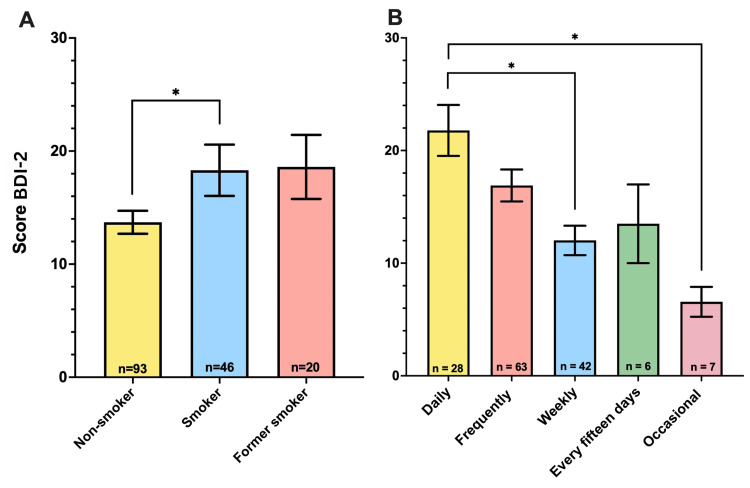
Influence of smoking and ultra-processed food consumption on depression (A) Comparison of BDI-2 scores among non-smokers (n=93), smokers (n=46), and former smokers (n=20) (F=3.72, p=0.026). Results show that smokers have a significantly higher BDI-2 score compared to non-smokers, suggesting a higher level of depression among smokers (non-smokers vs. smokers, p=0.048). The significant difference is indicated with an asterisk (*). (B) Comparison of BDI-2 scores based on the frequency of ultra-processed food consumption: daily (n=28), frequent (n=63), weekly (n=42), bi-weekly (n=6), and occasional (n=7) (F=5.38, p<0.001). Results indicate that participants who consume ultra-processed foods daily have a significantly higher BDI-2 score than those who consume them less frequently, suggesting a higher level of depression among frequent consumers (daily vs. weekly, p=0.002; daily vs. occasional, p=0.006). Significant differences are indicated with asterisks (*). BDI-2: Beck Depression Inventory-II

Smoking and frequent consumption of ultra-processed foods are associated with higher levels of depression. Smoking and frequent consumption of ultra-processed foods were associated with higher levels of depression. Smokers had significantly higher BDI-2 scores compared to non-smokers. Similarly, participants who consumed ultra-processed foods daily had higher BDI-2 scores than those who consumed them less frequently (Figure 5).

Relationship between depression categories and PSQI scores

**Figure 6:**
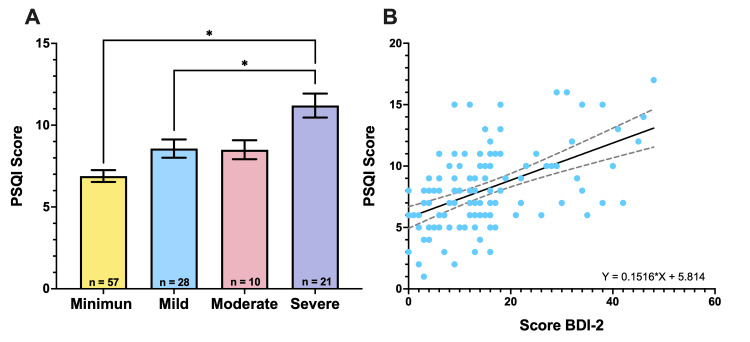
Comparison of PSQI scores according to BDI-2 depression categories (A) Comparison of PSQI scores across different depression categories according to the BDI-2 scale (minimal, mild, moderate, and severe) (F=11.8, p<0.001). The data show an increase in PSQI scores with the severity of depression, indicating poorer sleep quality in individuals with higher depression levels (minimal vs. severe, p<0.001; mild vs. severe, p=0.010). Significant differences are marked with asterisks (*). (B) Correlation between PSQI score and BDI-2 score. A positive correlation is observed, with a Spearman correlation coefficient of 0.5 (p<0.001), suggesting that higher BDI-2 scores are associated with higher PSQI scores, indicating an association between higher levels of depression and poorer sleep quality. The regression line and its 95% confidence interval are shown in the graph. PSQI: Pittsburgh Sleep Quality Index, BDI-2: Beck Depression Inventory-II

Individuals with higher levels of depression exhibit poorer sleep quality, and there is a positive correlation between depression scores and sleep quality scores. Individuals with higher levels of depression exhibited poorer sleep quality, and there was a positive correlation between depression scores (BDI-2) and sleep quality scores (PSQI), suggesting that higher levels of depression correspond to lower sleep quality indices (Figure 6).

## Discussion

In this study, various factors related to cardiovascular health, eating habits, physical activity, and their impact on depression and sleep quality were analyzed in a young adult population. The results indicate notable differences in compliance with cardiovascular health index components between men and women. Women tended to meet more adequate components of the health index than men (Figure 1). Specifically, a higher percentage of men meet three components, whereas a higher percentage of women meet seven adequate components. Additionally, women show greater adherence to fruit and vegetable consumption and physical activity, which may contribute to their better overall health profile than men. These findings are consistent with previous studies indicating a tendency for women to engage in more healthy behaviors, including greater adherence to diets rich in fruits and vegetables and higher levels of physical activity [28,29]. The literature suggests that these differences may be influenced by sociocultural and biological factors that affect lifestyle choices and health perceptions [30].

The analysis of healthy habits showed that higher consumption of fruits and vegetables, as well as higher levels of physical activity, were associated with lower levels of visceral fat (Figure 2). Participants with adequate fruit and vegetable consumption had significantly lower visceral fat levels than those with poor consumption. Similarly, those with ideal and moderate levels of physical activity had significantly lower visceral fat levels than those with low levels of physical activity. Previous research highlights the importance of a balanced diet and regular physical activity in reducing visceral fat [31,32]. A higher intake of fruits and vegetables and physical activity are inversely correlated with the accumulation of visceral fat, which contributes to the improvement of metabolic and cardiovascular health [33].

The data also revealed a significant correlation between visceral fat levels and various cardiovascular health parameters. Higher levels of visceral fat are associated with an increase in both systolic and diastolic blood pressure, indicating that the accumulation of visceral fat may contribute to a higher risk of hypertension. Conversely, there was a negative correlation between visceral fat and HDL cholesterol levels, suggesting that higher levels of visceral fat are associated with lower HDL cholesterol levels, which can increase the risk of cardiovascular disease (Figure 3). These results are consistent with those of previous studies that identified visceral fat as a key predictor of hypertension and dyslipidemia [34,35]. Literature supports the notion that visceral fat plays a significant role in the pathogenesis of cardiovascular diseases, mediated by its influence on insulin resistance, chronic inflammation, and alterations in the lipid profile [36].

The distribution of the nutritional status of the participants (Figure 4) showed that the majority of individuals fell into the normal-weight category (97%). However, a significant proportion of the population is overweight (45%) and obese (50%), highlighting the need for interventions to address these public health issues. The lower proportion of individuals in the overweight and obesity categories also indicates that most of the population maintains a weight within the recommended range. The prevalence of overweight and obesity observed in this study reflects global trends documented in numerous public health reports [37,38]. The increasing incidence of overweight and obesity underscores the urgency of implementing effective prevention and control strategies, focusing on nutritional education and promoting active lifestyles [39].

However, the impact of smoking and consumption of ultra-processed foods on depression scores (BDI-2) showed that both factors were associated with higher levels of depression (Figure 5). Smokers had a significantly higher BDI-2 score than non-smokers, indicating a higher level of depression among smokers. Similarly, participants who consumed ultra-processed foods daily had a significantly higher BDI-2 score than those who consumed them less frequently, suggesting a relationship between the frequent consumption of ultra-processed foods and higher levels of depression. This indicates that smoking and the consumption of ultra-processed foods are associated with an increased risk of depression [40,41]. Literature suggests that smoking can influence brain neurochemistry and inflammation patterns, contributing to depression [42]. Similarly, ultra-processed foods rich in sugars and trans fats are associated with an increase in depressive symptoms owing to their negative effects on metabolic and neuropsychological health [43].

Finally, individuals with higher levels of depression exhibited poorer sleep quality (Figure 6). Additionally, there was a positive correlation between depression scores (BDI-2) and sleep quality scores (PSQI), suggesting that higher levels of depression corresponded to worse sleep quality indices. The relationship between depression and sleep quality is well documented in the literature. Studies have demonstrated that sleep disorders are common among people with depression and that sleep quality deteriorates with the increasing severity of depression [44,45]. It is important to address both depression and sleep problems in clinical interventions to improve the mental health and overall health of individuals.

Limitations

The limitations of this study include the cross-sectional design, which prevents establishing causal relationships between the variables studied and limits the ability to observe changes over time. Additionally, the sample was obtained through non-probabilistic sampling, which may limit the generalizability of the results to other populations. The use of self-administered questionnaires may introduce reporting biases and errors in the measurement of health behaviors. The lack of control for confounding variables, such as genetics and socioeconomic environment, may influence the results obtained.

## Conclusions

These findings highlight the importance of a healthy diet and physical activity in maintaining good cardiovascular and mental health. Additionally, they demonstrated the adverse effects of smoking and consumption of ultra-processed foods on depression and sleep quality, indicating key areas for public health interventions. Furthermore, they reinforce the importance of promoting healthy behaviors and provide a foundation for developing interventions that address both the physical and mental health of the population.

## Appendices

**Table 1:** Health index components This table outlines the components of the health index, divided into health behaviors and health factors. Each component includes specific questions or measurements related to diet, physical activity, nicotine exposure, sleep, blood pressure, cholesterol, blood sugar, and body composition. These components are used to assess the overall health status of individuals.

Domain	Component	
Health behaviors	Diet	How many servings of vegetables do you consume per week? _____ servings 1, serving = 1/2 cup
		How many servings of red meat, hamburger, bacon, or sausage do you consume per week? _____ servings, 1 serving = 3 oz cooked
		How many servings of butter or cream do you consume per week? _____ servings, 1 serving = 1 tbsp
		How many servings of whole grains do you consume per day? _____ servings, 1 serving = 1 slice bread or 3/4 cup pasta or cereal
		How many times per week do you consume meals from fast food restaurants? ______number of times
		How many servings of fruit do you consume per week? _____ servings, 1 serving = 1/2 cup
		How many servings of fish or shellfish/seafood do you consume per week? ____ servings, 1 serving = 3 oz cooked, not fried
		How many servings of beans do you consume per week? _____ servings, 1 serving = 1/2 cup
		How many servings of commercial sweets, candy bars, pastries, cookies, or cakes do you consume per week? ______ servings, 1 serving = 1 item, bar or slice
		How many servings of sugar-sweetened beverages do you drink per week? _____ servings, 1 serving = 12 oz (do not include diet soda)
	Physical activity	How many minutes of moderate (or greater) intensity activity per week? ____ minutes
	Nicotine exposure	What is your smoking status? Select: current smoker or former smoker, quit <1 year or currently using inhaled NDS; former smoker, quit 1-<5 years; former smoker, quit ≥5 years; never smoked; I live with an active indoor smoker
	Sleep	On average, how many hours of sleep do you get in a 24-hour period? ____ hours
Health factors	Blood pressure	Systolic ____ mmHg, diastolic____ mmHg, select if applicable: I take medication to lower my blood pressure
	Cholesterol	Total cholesterol ____ mg/dL, HDL cholesterol ____mg/dL, select if applicable: I take medication to lower my cholesterol
	Blood sugar	Fasting _____mg/dL, select if applicable: I have diabetes (either type 1 or 2)
	Body composition	Height ___ m ___ cm, weight ___ Kg

**Table 2:** PSQI Self-assessment questionnaire that measures sleep quality in seven components, evaluating one month of sleep. Higher scores indicate poorer sleep quality. PSQI: Pittsburgh Sleep Quality Index

1. During the past month, when have you usually gone to bed at night?	Usual bedtime			
2. During the past month, how long (in minutes) has it usually taken you to fall asleep each night?	Number of minutes			
3. During the past month, when have you usually gotten up in the morning?	Usual getting up time			
4. During the past month, how many hours of actual sleep did you get at night? (This may be different than the number of hours you spend in bed.)	Hours of sleep per night			
5. During the past month, how often have you had trouble sleeping because you.				
Cannot get to sleep within 30 minutes	Not during the past month	Less than once a week	Once or twice a week	Tree or more times a week
(b) Wake up in the middle of the night or early morning	Not during the past month	Less than once a week	Once or twice a week	Tree or more times a week
(c) Have to get up to use the bathroom	Not during the past month	Less than once a week	Once or twice a week	Tree or more times a week
(d) Cannot breathe comfortably	Not during the past month	Less than once a week	Once or twice a week	Tree or more times a week
(e) Cough or snore loudly	Not during the past month	Less than once a week	Once or twice a week	Tree or more times a week
(f) Feel too cold	Not during the past month	Less than once a week	Once or twice a week	Tree or more times a week
(g) feel too hot	Not during the past month	Less than once a week	Once or twice a week	Tree or more times a week
(h) Has bad dreams	Not during the past month	Less than once a week	Once or twice a week	Tree or more times a week
(I) Have pain	Not during the past month	Less than once a week	Once or twice a week	Tree or more times a week
(j) Other reason(s) please describe	Not during the past month	Less than once a week	Once or twice a week	Tree or more times a week
6. During the past month, how would you rate your sleep quality overall?	Very good	Fairly good	Fairly bad	Very bad
7. During the past month, how often have you taken medicine (prescribed or “over the counter”) to help you sleep?	Not during the past month	Less than once a week	Once or twice a week	Tree or more times a week
8. During the past month, how often have you had trouble staying awake while driving, eating meals, or engaging in social activity?	Not during the past month	Less than once a week	Once or twice a week	Tree or more times a week
9. During the past month, how much of a problem has it been for you to keep up enough enthusiasm to get things done?	No problem at all	Only a very slight problem	Somewhat of a problem	A very big problem
10. Do you have a bed partner or roommate?	No bed partner or roommate	Partner/ roommate in other room	Partner in the same room, but not same bed	Partner in the same bed
If you have a roommate or bed partner, ask him/her how often in the past month you have had.				
(a) Loud snoring	Not during the past month	Less than once a week	Once or twice a week	Tree or more times a week
(b) Long pauses between breaths while asleep	Not during the past month	Less than once a week	Once or twice a week	Tree or more times a week
(c) Legs twitching or jerking while you sleep	Not during the past month	Less than once a week	Once or twice a week	Tree or more times a week
(d) Episodes of disorientation or confusion during sleep	Not during the past month	Less than once a week	Once or twice a week	Tree or more times a week
(e) Other restlessness while you sleep, please describe	Not during the past month	Less than once a week	Once or twice a week	Tree or more times a week

**Table 3:** BDI Self-assessment questionnaire that measures the severity of depression. It evaluates emotional, cognitive, and physical symptoms. Higher scores indicate a greater severity of depression. BDI: Beck Depression Inventory

Mood	0 I do not feel sad	1 I feel blue or sad	2a I am blue or sad all the time and I can't snap out of it	2b I am so sad or unhappy that it is very painful	3 I am so sad and unhappy that I can't stand it
B. Pessimism	0 I am not particularly discouraged about the future	1a I feel discouraged about the future	2a I feel I have nothing to look forward to	2b I feel that I won't ever get over my troubles	3 I feel the future is hopeless and that things cannot improve
C. Sense of failure	0 I do not feel like a failure	1 I feel I have failed more than the average person	2a As I look back on my life, all I can see is a lot of failures	2b As I look bacon my life all I can see is a lot of failures	3 I feel I am a complete failure as a person
D. Lack of satisfaction	0 I get as much satisfaction out of things as I used to	1a I don't enjoy things the way I used to	2b I don't get real satisfaction out of anything anymore	3 I am dissatisfied or bored with everything	
E. Guilty feeling	0 I don't feel particularly guilty	1 I feel bad or unworthy a good part of the time	2a I feel quite guilty	2a I feel bad or unworthy practically all the time now	3 I feel guilty all of the time
F. Sense of punishment	0 I don't feel I am being punished	1 I have a feeling that something bad may happen to me	2 I feel I am being punished or will be punished	3a I feel I deserve to be punished	3b I want to be punished
G. Self hate	0 I don't feel disappointed in myself	1a I am disappointed in myself	lb I don't like myself	2 I am disgusted with myself	3 I hate myself
H. Self accusations	0 I don't feel I am any worse than anybody else	1 I am critical of myself for my weaknesses or mistakes	2a I blame myself for everything that goes wrong	2b I feel I have many bad faults	
I. Self-punitive wishes	0 I don't have any thoughts of harming myself	1 I have thoughts of harming myself, but I would not carry them out	2a I feel I would be better off dead	2b I have definite plans about commit ting suicide. 2c I feel my family would be better off if I were dead	3 I would kill myself if I could
J. Crying spells	0 I don't cry any more than usual	1 I cry more now than I used to	2 I cry all the time now. I can´t stop it	3 I used to be able to cry but now I can't cry at all even though I want to	
K. Irritability	0 I am no more irritated now than I ever am	1 I get annoyed or irritated more easily than I used to	2 I feel irritated all the time	3 I don´t get irritated at all at the things that uses to irritate me	
L. Social withdrawal	0 I have not lost interest in other people	1 I am less interested in other people than I used to be	2 I have lost most of my interest in other people and have little feeling for them	3 I have lost all of my interest in other people and don't care about them at all	
M. Indecisiveness	0 I make decisions about as well as I ever	1I am less sure of myself now and try to put off making decisions	2 I can't make decisions any more without help	3 I can't make any decisions at all any	
N. Body image	0 I don't feel I look any worse than I used to	1 I am worried that I am looking old or unattractive	2 I feel that there are permanent changes in my appearance that make me look unattractive	3 I feel that I am ugly or repulsive looking	
O. Work inhibition	0 I can work about as well as before	1 It takes an extra effort to get started at doing something. lb I don't work as well as I used to	2 I have to push myself very hard to do anything	3 I can't do any work at all	
P. Sleep disturbance	0 I can sleep as well as usual	1 I wake up more tired in the morning than I used to	2 I wake up 1-2 hours earlier than usual and find it hard to get back to sleep	3 I wake up early every day and can't get more than 5 hours sleep	
Q. Fatigability	0 I don't get more tired than usual	1 I get tired more easily than I used to	2 I get tired from doing almost anything	3 I am too tired to do anything	
R. Loss of apettite	0 My appetite is no worse than usual	1 My appetite is not as good as it used to be	2 My appetite is much worse now	3 I have no appetite at all anymore	
S. Weight loss	0 I haven't lost much weight, if any, lately.	1 I have lost more than five pounds	2 I have lost more than ten pounds	3 I have lost more than fifteen pounds	
T. Somatic preoccupation	0 I am no more worried about my health than usual	1 I am concerned about aches and pains or upset stomach or constipation or other unpleasant feelings in my body	2 I am so concerned with how I feel or what I feel that it's hard to think of much else	3 I am completely absorbed in what I feel	
U. Loss of libido	0 I have not noticed any recent change in my interest in sex	1 I am less interested in sex than I used to be	2 I am much less interested in sex now	3 I have lost interest in sex completely	
